# Neuroprotective Mechanisms of PPAR**δ**: Modulation of Oxidative Stress and Inflammatory Processes

**DOI:** 10.1155/2011/373560

**Published:** 2011-10-29

**Authors:** Caroline I. Schnegg, Mike E. Robbins

**Affiliations:** ^1^Department of Cancer Biology, Comprehensive Cancer Center, Wake Forest School of Medicine, Winston-Salem, NC 27157, USA; ^2^Brain Tumor Center of Excellence, Comprehensive Cancer Center, Wake Forest School of Medicine, Winston-Salem, NC 27157, USA; ^3^Department of Radiation Oncology, Comprehensive Cancer Center, Wake Forest School of Medicine, Medical Center Boulevard, Winston-Salem, NC 27157, USA

## Abstract

Peroxisome proliferator-activated receptors (PPAR**α**, **δ**, and **γ**) are ligand-activated transcription factors that regulate a wide range of cellular processes, including inflammation, proliferation, differentiation, metabolism, and energy homeostasis. All three PPAR subtypes have been identified in the central nervous system (CNS) of rodents. While PPAR**α** and PPAR**γ** are expressed in more restricted areas of the CNS, PPAR**δ** is ubiquitously expressed and is the predominant subtype. Although data regarding PPAR**δ** are limited, studies have demonstrated that administration of PPAR**δ** agonists confers neuroprotection following various acute and chronic injuries to the CNS, such as stroke, multiple sclerosis, and Alzheimer's disease. The antioxidant and anti-inflammatory properties of PPAR**δ** agonists are thought to underly their neuroprotective efficacy. This review will focus on the putative neuroprotective benefits of therapeutically targeting PPAR**δ** in the CNS, and specifically, highlight the antioxidant and anti-inflammatory functions of PPAR**δ** agonists.

## 1. Introduction

Peroxisome proliferator-activated receptors (PPARs) are members of the nuclear hormone receptor superfamily. Three closely related subtypes have been identified—PPAR*α* (NR1C1), PPAR*δ* (NR1C2, PPAR*β*), and PPAR*γ* (NR1C3) [1]. They are ligand-activated transcription factors that heterodimerize with the retinoid X receptor to regulate transcription [2, 3]. Each PPAR subtype is encoded by a different gene and has a unique tissue distribution. PPARs possess a high degree of structural homology and share the same DNA response element, termed the PPAR response element (PPRE). PPREs consist of an AGGTCA hexameric direct repeat separated by one or two nucleotides [4]. Despite sharing PPREs, the three subtypes have both similar and different functions. The specificity of their functions appears to depend on their differential (i) tissue expression, (ii) ligand specificity, (iii) A/B-domain, (iv) posttranslational modifications, (v) affinity for cofactors, (vi) affinity for individual PPREs, and (vii) nongenomic actions [5]. 

A common function of the PPAR subtypes is suppressing oxidative stress and inflammatory processes [6]. Although the CNS was once thought to be immune privileged due to the protection of the BBB, numerous studies have demonstrated that inflammation does occur in the brain and spinal cord and is associated with many neurodegenerative disorders [7]. These findings provided the original rationale for assessing the neuroprotective efficacy of PPAR agonists in a variety of CNS disorders. The neuroprotective efficacy of PPAR*α* and PPAR*γ* agonists has been reviewed previously [8–10].

Despite being the predominant subtype in the CNS [11], data regarding the neuroprotective efficacy of PPAR*δ* agonists are limited compared to PPAR*α* or PPAR*γ* agonists. This likely reflects the fact that PPAR*δ*-specific agonists and antagonists have only recently become available. Several studies have used the synthetic PPAR*δ*-specific agonists L-165041, L-160,043, GW0742, or GW501516 to explore the functional activities of the receptor [12–15]. Recent studies assessing the neuroprotective efficacy of synthetic PPAR*δ* agonists indicate that they may ameliorate clinical symptoms and reduce the severity of a variety of acute and chronic CNS pathologies, in large part, by modulation of oxidative stress and inflammatory responses associated with the diseases. Recent reports suggest that PPAR*δ* may have additional protective effects against the progression of CNS disorders, such as promoting cell survival [12, 13, 16]. To date, the neuroprotective benefits of PPAR*δ* agonists have been observed in models of stroke, multiple sclerosis, Alzheimer's disease, Parkinson's disease, radiation-induced brain injury, and spinal cord injury [12, 17–23]. 

## 2. PPAR*δ* Expression in the CNS

The expression of PPAR*δ* has been examined in many rodent tissues, including the CNS. The receptor is thought to be ubiquitously expressed in the adult rodent brain and spinal cord [11, 24–26]. In rats, PPAR*δ* begins to be expressed at midgestation, reaching peak expression in all neural tissues in the late stage embryonic brain, suggesting that it may play a role in cell differentiation in the CNS [27]. In situ hybridization and immunolocalization studies have demonstrated that high levels of PPAR*δ* are found in the hippocampus, telencephalic cortex, and the cerebellar cortex of rats [11, 27, 28]. Other studies in the rat brain indicate that PPAR*δ* mRNA is highly expressed in the thalamic nuclei [25]. In the rat spinal cord, PPAR*δ* is the most abundant PPAR subtype; it is expressed in every cell layer and highly expressed in the lamina II and lamina IX layers [11]. Rat brain endothelial cells also express PPAR*δ* mRNA, suggesting a possible role in blood-brain barrier (BBB) maintenance [29]. PPAR*δ* is also widely expressed in the mouse brain, with high levels in the entorhinal cortex, hypothalamus, hippocampus, and corpus callosum. Localization studies identified PPAR*δ* mRNA and protein in mouse neurons and oligodendrocytes, but not in astrocytes [26]. However, in cell culture, PPAR*δ* expression has been documented in mouse and rat cortical astrocytes as well as in rat cerebral astrocytes [30, 31]. Results from our own laboratory demonstrate that the receptor is also expressed in microglia. Taken together, PPAR*δ* expression has been observed in all the major cell types within the CNS, including astrocytes, endothelial cells, microglia, neurons, and oligodendrocytes.

## 3. Stroke-Induced Brain Injury

Strokes are often caused by a blockage of blood flow in the brain, referred to as ischemia, which results in a shortage of oxygen to the brain. Vascular degradation after cerebral ischemia leads to BBB disruption and neuronal loss. Of the three PPAR isotypes, PPAR*δ* is the highest expressed in the brain's parenchyma and cerebral vasculature, and increasing evidence suggests that PPAR*δ* may protect against ischemic insults [13]. This was first highlighted in PPAR*δ*-null mice, which compared to WT mice, had a significant increase in infarct size after focal cerebral ischemia [17, 18]. The difference in infarct size was detected as early as 30 min after induction of ischemia, suggesting that PPAR*δ* plays an early role in protection [17]. Following middle cerebral artery occlusion (MCAO), increased cerebrovascular permeability and infarct size were detected in mice with specific deletion of PPAR*δ* in vascular smooth muscle cells (VSMCs) [13]. Additionally, PPAR*δ* agonists appear to be protective after cerebral ischemia; rats given infusions of L-165041 or GW501516 had significantly attenuated ischemic damage 24 h after MCAO [12]. The mechanisms underlying the protective effect of PPAR*δ*, however, are unclear. Potential protective effects include (i) modulating oxidative stress and proinflammatory responses, (ii) maintaining matrix-cell adhesions, and (iii) promoting cell survival.

The enhanced oxidative stress and inflammatory responses in an ischemic brain are thought to contribute to neuronal death. The antioxidant and anti-inflammatory actions of PPAR*δ* agonists have been observed in a variety of cell types, including astrocytes and microglia [32–35]. In particular, PPAR*δ* can activate transcription of antioxidant genes, including catalase and superoxide dismutase (SOD) [36–38]. PPAR*δ* can also control gene transcription independent of binding to PPREs. Transrepression is thought to underlie many of the anti-inflammatory effects of PPAR*δ*. PPAR*δ* can exert transrepression by directly interacting with nuclear factor-*κ*B (NF-*κ*B), thereby inhibiting activation of the proinflammatory transcription factor [38]. In addition, PPAR*δ* exerts transrepression in macrophages by relocating the transcriptional repressor B-cell lymphoma protein 6 (Bcl-6) from PPAR*δ* to the promoter regions of proinflammatory genes, including the vascular cell adhesion molecule-1 (VCAM-1) and the monocyte chemoattractant protein-1 (MCP-1) [39–41].

It is possible that modulation of these processes contributes to the protective effect of PPAR*δ* after cerebral ischemia. In an *in vivo* model of MCAO, for example, there was an increase in the lipid peroxidation marker, malondialdehyde, and a decrease in the levels of the antioxidant, glutathione, and the antioxidant enzyme, manganese (Mn)SOD, in PPAR*δ*-null mice compared to WT mice. This suggests that there is increased oxidative stress in PPAR*δ*-null mice following cerebral ischemia [18]. Additionally, mice with PPAR*δ* specifically deleted in their VSMCs had a significant increase in proinflammatory mediators in their brain 24 h after MCAO. This included an increase in the mRNA levels of interleukin-1 beta (IL-1*β*), IL-6, intercellular adhesion molecule-1 (ICAM-1), and MCP-1 [13]. Pioglitazone, a PPAR*γ* agonist, has been shown to confer neuroprotection following MCAO. This is thought to occur, in part, by activation of PPAR*δ*, which enhances production of the IL-1 receptor antagonist (IL-1Ra). IL-1Ra promotes neuroprotection by competing with binding of IL-1*β* to IL-1R1; consequently, this represses receptor signaling and reduces inflammatory responses following ischemia [42]. 

The pathogenesis of cerebral ischemia also involves the breakdown of matrix-cell adhesions, which results in the disruption of the BBB and ultimately neuronal death and brain damage. PPAR*δ* may regulate these responses by decreasing activity of matrix metalloproteinases (MMPs), enzymes that degrade structural proteins in the extracellular matrix and that have been implicated in ischemia-induced parenchymal and vascular damage. In particular, treatment of VSMCs with the PPAR*δ* agonist, GW501516, reduced MMP-9 enzymatic activity 24 h after oxygen-glucose deprivation (OGD). Furthermore, VSMC-selective PPAR*δ* knockout mice exhibited increased MMP-9 expression following MCAO, and inhibition of MMP-9 using shRNA reduced infarct size and vascular permeability in these mice [13]. More studies, however, are needed to understand the mechanism by which PPAR*δ* regulates MMP-9 expression.

PPAR*δ* may also promote cell survival after ischemic insults. In particular, GW501516 significantly reduced cerebral vascular endothelial cell degeneration in an OGD mouse model. GW501516 protected against vascular cell death by inhibiting expression of miR-15a, which directly regulates the antiapoptotic protein Bcl-2. This resulted in increased Bcl-2 protein expression, reduced Golgi fragmentation, and decreased caspase-3 activity, which in turn reduced cerebrovascular permeability and infarct volume *in vivo* [43]. Although not tested with respect to protection against stroke-induced injury, PPAR*δ* has been shown to promote survival of neurons under stress conditions. Specifically, L-165041 or GW501516 significantly reduced SH-SY5Y cell death after exposure to (i) thapsigargin, an endoplasmic reticulum Ca2+ ATPase inhibitor; (ii) 1-methyl-4-pheylpyridinium (MPP+), a dopaminergic neurotoxin; or (iii) staurosporine, a protein kinase inhibitor. The effect on viability may be due to inhibition of apoptosis, as both agonists could significantly attenuate caspase-3 and caspase-7 activity in cells treated with one of the three cytotoxins [12]. GW0742 has also been shown to be neuroprotective in primary cultures of rat cerebellar granule neurons exposed to low-KCl media. Prolonged exposure of primary cultures to GW0742, however, induced apoptotic cell death [44]. Future studies should examine if, similar to vascular endothelial cells, PPAR*δ* agonists can also promote neuronal survival after ischemic insults.

## 4. Multiple Sclerosis

Multiple sclerosis (MS) is an autoimmune disease that results in the expansion of pathogenic T cells specific for myelin autoantigens [45]. The disease manifests in the destruction of myelin sheaths and the inflammatory activation of glial cells [46]. Experimental autoimmune encephalomyelitis (EAE), in which mice are immunized with the encephalitogenic myelin oligodendrocyte glycoprotein (MOG) peptide, is used as a model of MS [47, 48]. The potential role of PPAR*δ* in demyelinating diseases was first proposed in a model that attempted to explain the detrimental effects of inflammatory mediators on myelin synthesis. Specifically, tumor necrosis factor alpha (TNF-*α*), a cytokine implicated in demyelinating pathologies, was demonstrated to attenuate PPAR*δ* expression in OPCs and, concurrently, decrease very long-chain fatty acid (VLCFA) *β*-oxidation. The hypothetical model proposed that decreased PPAR*δ* expression would lead to VLCFA accumulation, which would induce cytotoxic effects and demyelination. PPAR*δ* agonists, therefore, were postulated to be beneficial for MS-affected individuals [49].

GW0742 was the first PPAR*δ* agonist to show beneficial effects in a mouse model of EAE [14, 20]. The effects of GW0742 were greater when administered to mice during disease progression, rather than when given at the time of MOG administration. Specifically, GW0742 reduced the emergence of new cortical lesions, increased proteolipid protein (PLP) mRNA levels, and reduced IL-1*β* levels [14]. The levels of interferon-gamma (IFN*γ*) were not altered by GW0742, which is in contrast to observations in mouse and human immune cells that demonstrate GW0742 can inhibit production of IFN-*γ* [14, 20]. The protective effects of GW0742 are likely mediated by reducing inflammatory cell activation [14].

Research indicates that GW501516 and L-165041 are also effective at ameliorating EAE. In mice, the attenuation of EAE by GW501516 or L-165041 was associated with decreased expression of the proinflammatory cytokines, IL-2 and IL-23, and increased expression of the anti-inflammatory cytokines, IL-4 and IL-10. Reduced production of IFN*γ* and IL-17 by T helper type 1 (Th1) and Th17 cells was also observed in EAE mice treated with GW501516 or L-165041 [50]. In accord with these findings, PPAR*δ*-null mice with EAE had impaired Th1/Th17 and Th2/Treg responses, which may contribute to the extended recovery time in PPAR*δ*-null mice compared to WT mice. The prolonged EAE in PPAR*δ*-null mice was associated with sustained levels of IFN*γ*, IL-17, IL-12p35, and IL-12p40, consistent with the hypothesis that PPAR*δ* regulates inflammatory responses associated with EAE [19]. 

PPAR*δ*-null mice with EAE also exhibited a severe inflammatory response in their spinal cord—they had a higher frequency of CD4+ cells that had an accumulation of IFN-*γ* or IL-17A compared to WT mice [20]. Intriguingly, PPAR*δ* also appears to play a protective role in the spinal cords of steroid-receptor-coactivator-3-(SRC-3-) null mice with EAE. SRC-3-null mice had a significantly reduced severity of EAE that was associated with diminished inflammatory responses and decreased demyelination. The attenuated EAE symptoms in SRC-3-null mice are thought to occur via upregulation of PPAR*δ*. SRC-3-null mice displayed decreased expression of the proinflammatory markers, TNF-*α*, IFN-*γ*, CCL2, CCL3, CCL5, and CXCL10, and increased expression of the anti-inflammatory markers, IL-10 and opsonins. Myelin genes—myelin basic protein (MBP) and PLP—were also significantly higher in SRC-3-null mice, which may be a result of increased PPAR*δ* expression [51]. 

In SRC-3 mice, PPAR*δ* expression is thought to alternatively activate microglia from the proinflammatory M1 phenotype to the anti-inflammatory M2 phenotype. In particular, there was a correlation between PPAR*δ* expression in SRC-3-null mice and ramified microglia that expressed Ym1/2 and Mrc1, phenotypic markers for alternative activation. Interestingly, PPAR*δ* activation has been shown to control the alternative activation of adipose tissue macrophages (ATMs) and Kupffer cells, hepatic macrophages [52, 53]. In the white adipose tissue of PPAR*δ*-null mice, genes of alternatively activated macrophages, Clec7a, Retnla, Tgfb1, Jag1, and Mrc1, were reduced. The alternative activation of Kupffer cells was also decreased in PPAR*δ*-null mice, as assessed by reduced expression of Arg1, Clec7a, Jag1, Pdcd1lg2, and Chia [53]. Reduced alternative activation of ATMs and Kupffer cells in PPAR*δ*-null mice led to impaired glucose tolerance and insulin resistance [52, 53]. Whether PPAR*δ* agonists can alternatively activate microglia remains to be determined. 

To further examine the mechanisms by which PPAR*δ* is protective in models of EAE, studies have used *in vitro* models of aggregating brain cells. GW501516 was effective at reducing IFN-*γ*- or lipopolysaccharide- (LPS-) induced TNF-*α* or inducible nitric oxide synthase (iNOS) mRNA levels; however, it increased IL-6 mRNA expression. In the presence of anti-MOG, a demyelinating antibody, GW501516 was unable to prevent decreases in MBP [54]. These results further suggest that in models of EAE, the protective effects of PPAR*δ* agonists may be due to their anti-inflammatory properties rather than their effects on myelin genes or oligodendrocytes. Collectively, the findings above indicate that PPAR*δ* agonists ameliorate the inflammatory responses in EAE and may represent therapeutic avenues to ameliorate MS progression.

## 5. Alzheimer's Disease

Alzheimer's disease (AD) is a neurodegenerative disorder characterized by neuronal degeneration and progressive cognitive impairment [55]. While the pathogenesis of AD is not fully understood, AD patients exhibit an accumulation of amyloid plaques formed by oligomerization of the amyloidogenic peptides, A*β* 1–42 and A*β* 1–40, and neurofibrillary tangles formed by tau protein. These plaques and tangles are thought to contribute to neurodegeneration [56]. Inflammatory cytokines may lead to an increase in A*β* peptides, thereby contributing to the progression of AD. The neurotransmitter noradrenaline (NA) can protect neurons from various inflammatory stimuli, including exposure to A*β* peptides. This is thought to occur, in part, by activation of PPAR*δ*. In particular, in primary cultures of rat cortical neurons, A*β* peptides induced cell death as measured by LDH release. The ability of NA to prevent A*β* peptide-induced cell death was decreased in the presence of GW9662, a PPAR antagonist. Moreover, GW0742 blocked cell death to the same degree as NA, as measured by LDH release and Fluoro-Jade B staining [21]. 

Further supporting the possible benefits of targeting PPAR*δ* in the treatment of AD, administration of GW0742 significantly reduced amyloid plaque burden in the subiculum of 5xFAD mice. These mice overexpress the amyloid precursor protein and presenilin 1, display plaques and inflammation, and develop neuronal damage and cognitive impairment. Interestingly, the decrease in amyloid burden in GW0742 treated 5xFAD mice was associated with increased expression of neprilysin, an amyloid-degrading enzyme. Furthermore, GW0742 could activate a neprilysin promoter driving luciferase expression *in vitro*. GW0742 also led to a decrease in astrocyte activation in the 5xFAD mice, as assessed by GFAP staining [22]. 

Streptozotocin (STZ) administered to mice intracerebrally depletes brain insulin levels and induces progressive neurodegeneration that corresponds to the clinical symptoms of AD. Impairments in insulin and insulin-like growth factor signaling are observed in AD brains and these abnormalities increase concomitantly with dementia [57, 58]. Treatment with the PPAR*δ* agonist, L-160,043, prevented STZ-induced neurodegeneration and ameliorated cognitive impairment as evaluated by the Morris Water Maze task. These effects of L-160,043 were attributed to (i) increased insulin receptor signaling, (ii) reduced tau phosphorylation, (iii) increased choline acetyltransferase, and (iv) attenuated inflammation and oxidative stress. Consistent with the effects of GW0742 described above, L-160,043 reduced levels of GFAP. Furthermore, L-160,043 reduced immunoreactivity of the oxidative stress markers, 8-hydroxyguanosine (8-OHdG) and 4-hydroxynonenal (HNE), relative to STZ-treated animals [15]. Elevated levels of 8-OHdG and HNE have been detected in AD brains [59, 60]. This suggests that PPAR*δ* agonists may be efficacious antioxidant agents to ameliorate oxidative stress associated with AD. Interestingly, the actions of L-160,043 were more pronounced than the PPAR*α* agonist, GW7647, or partial PPAR*γ* agonist, F-L-Leu [15]. It is important to note that the PPAR*δ* agonist was administered the same day as STZ. Future studies should assess whether PPAR*δ* activation at later stages of AD progression can also halt AD development.

## 6. Parkinson's Disease

PPAR*δ* agonists may exert therapeutic benefits in patients with Parkinson's disease (PD), a neurodegenerative disorder characterized by loss of dopaminergic neurons in the substantia nigra. Progression of PD has been attributed to reactive oxygen species (ROS) and oxidized dopamine, which are toxic to dopaminergic neurons [61]. This disease manifests itself in motor dysfunctions and tremors. The synthetic opiate 1-methyl-4-phenyl-1,2,3,6-tetrahydrodropyridine (MPTP) induces PD in drug-addicted individuals. In mice, administration of GW501516 or L-165041 48 h before the first injection of MPTP significantly ameliorated depletion of striatal dopamine and its metabolites. As mentioned above, these PPAR*δ* agonists also protected human neuroblastoma cells, SH-SY5Y, from cell death induced by the dopaminergic neurotoxin MPP+. This protection was associated with a significant reduction of caspase-3 and may contribute to the neuroprotective efficacy of GW501516 and L-165041 in experimental models of PD [12]. The protective role of PPAR*δ* in PD has not been adequately explored, and it is unclear if, similar to the PPAR*γ* agonist pioglitazone, PPAR*δ* agonists can modulate oxidative stress and inflammatory processes associated with PD. In particular, in a mouse model of PD, pioglitazone prevented MPTP-induced glial cell activation and loss of dopaminergic neurons. Mechanistically, neuronal death was prevented by inhibiting activation of the proinflammatory transcription factor NF-*κ*B by (i) increasing I*κ*-B*α* expression and (ii) inhibiting nuclear translocation of p65. Pioglitazone also decreased the number of GFAP and iNOS positive cells in both the striatum and substantia nigra pars compacta [62].

## 7. Astrogliosis

Many CNS disorders are associated with astrogliosis. During astrogliosis, astrocytes are thought to increase production of proinflammatory mediators, such as arachidonic acid [63, 64]. Arachidonic acid, which is a substrate for cyclooxygenase-1 (Cox-1) and Cox-2, is released by phospholipase A_2_ (PLA_2_). PLA_2_ and Cox-2 have been associated with neurodegenerative diseases [65, 66]. Interestingly, both secretory PLA_2_ (sPLA_2_) and cytosolic PLA_2_ (cPLA_2_) contain a PPRE in their promoters. PPAR*δ* expression appears to be abundant in immature cultures of primary rodent astrocytes [30]. In primary rat brain astrocytes, pretreatment of astrocytes with L-165041 attenuated LPS-induced sPLA_2_ expression, while astrocytes treated simultaneously with L-165041 and LPS displayed increased cPLA_2_ expression and had no change in sPLA_2_ expression. As the authors noted, LPS stimulates NF-*κ*B and activator protein-1 (AP-1), which PPARs have been shown to transrepress. It is possible that in this model LPS-induced activation of NF-*κ*B and AP-1 is acting to transrepress PPAR*δ* [35]. Furthermore, LPS-induced Cox-2 expression in rat primary astrocytes may be modulated, in part, by PPAR*δ*. In particular, rosiglitazone, a PPAR*γ* agonist, increased PPAR*δ* expression in cortical astrocytes and led to increased Cox-2 expression. On the other hand, GW7647, a PPAR*α* agonist, reduced PPAR*δ* levels and decreased LPS-induced Cox-2 expression [67]. These results are surprising given that studies largely focus on the antioxidant and anti-inflammatory properties of PPARs.

## 8. Radiation-Induced Brain Injury

Fractionated partial or whole-brain irradiation (fWBI) is often required to treat both primary and metastatic brain cancer. Radiation-induced brain injury, including progressive cognitive impairment, however, can significantly affect the well-being of the approximately 200,000 patients who receive these treatments each year [68, 69]. Recent reports indicate that the pathogenesis of radiation-induced brain injury may be caused, in part, by chronic oxidative stress and inflammatory responses, as well as increased microglial activation in the brain [70–72]. Microglia, which are termed the macrophages of the brain, are considered to be one of the key mediators of neuroinflammation [73–75]. Data suggest that irradiating microglial cells *in vitro* leads to an increase in a variety of proinflammatory mediators, including the cytokines, TNF-*α* and IL-1*β*, and the chemokines, MCP-1 and ICAM-1 [76–78]. Studies performed *in vivo* indicate that there is an increase in proinflammatory mediators within hours of irradiating the rodent brain [76, 79]. To date, studies in rodents have demonstrated that administration of anti-inflammatory drugs can decrease radiation-induced microglial activation [80, 81]. These findings provide a strong rationale for investigating whether administration of PPAR*δ* agonists can confer neuroprotection following irradiation.

Ongoing studies in our laboratory are investigating the ability of PPAR*δ* to modulate radiation-induced brain injury. Incubating BV-2 murine microglial cells with the PPAR*δ* agonist, L-165041, is effective at modulating radiation-induced injury, including inhibiting the radiation-induced increase in (i) intracellular ROS generation, (ii) Cox-2, iNOS and MCP-1 expression, (iii) IL-1*β* and TNF-*α* message levels, and (iv) NF-*κ*B and AP-1 activation (unpublished results). These observations demonstrate the important antioxidant and anti-inflammatory actions of PPAR*δ* agonists in microglial cells following irradiation and are similar to previous findings indicating that the PPAR*α* agonists, GW7647 and fenofibrate, can inhibit radiation-induced inflammatory markers in BV-2 cells [82]. 

Our laboratory has also assessed the anti-inflammatory actions of PPAR*δ* following irradiation *in vivo*. One week following a single dose of 10 Gy ^137^Cs *γ* WBI, young adult male C57Bl/6 mice exhibited a significant increase in the number of activated microglia (CD68^+^ cells) in the dentate gyrus (DG). GW0742 administered prior to, during, and after WBI prevented this radiation-induced increase in activated microglia. Interestingly, radiation did not induce an increase in activated microglia in the DG of PPAR*δ*-null mice at one week or two months after WBI. These results suggest that PPAR*δ* deficiency leads to an inhibition of microglial activation (unpublished results). These findings are surprising and quite different from those observed in PPAR*α*-null mice, where PPAR*α* deficiency resulted in a sustained increase in activated microglia seen at one week and two months after WBI [81]. This indicates that the effect of PPAR deficiency is subtype-dependent. 

Although PPAR*δ*-null mice do not show a radiation-induced microglial response, they may display an astrocytic response. Of note, PPAR*δ*-null mice express increased mRNA levels of GFAP, a marker of astrocytes, compared to WT mice. Additionally, while WBI reduced the number of astrocytes, GFAP^+^ cells, two months after irradiation in WT mice, there was no apparent decrease in the number of astrocytes in the irradiated PPAR*δ*-null mice. While these findings are preliminary, they do suggest that PPAR*δ* may modulate the radiation response of the astrocyte (unpublished results).

Although studies have not examined if PPAR*δ* agonists can modulate radiation-induced cognitive impairment, data demonstrate that PPAR*γ* can ameliorate or prevent radiation-induced cognitive decline. In particular, pioglitazone ameliorated radiation-induced cognitive impairment when administered prior to, during, and for 4 or 54 weeks after fWBI of young adult male rats [83]. While these results are promising, there are concerns with using pioglitazone in the clinic, including an increased risk of (i) weight gain and (ii) myocardial infarctions [84]. It is important to investigate if, similar to PPAR*γ*, PPAR*δ* can modulate radiation-induced cognitive impairment. Since PPAR*δ* is the primary subtype in the CNS, it is possible that PPAR*δ* agonists can mediate a more pronounced antioxidant and anti-inflammatory response in the brain.

## 9. Spinal Cord Injury

Spinal cord injury (SCI) is associated with altered conduction of axons and motor dysfunction due to oligodendrocyte and neuronal cell death [85]. SCI is accompanied by inflammation and edema, which results in infiltration of inflammatory cells, release of proinflammatory factors, vascular permeability, and ultimately necrosis [86, 87]. Data suggest that PPAR*δ* can modulate these responses. Administration of GW0742 to mice after SCI significantly improved their motor function. The PPAR*δ* antagonist, GSK0660, blocked the effect of GW0742 on motor function suggesting that the protective effects of the agonists are dependent on activation of the PPAR*δ* receptor. The effects of GW0742 were associated with reduced oxidative stress and proinflammatory responses in the spinal cord. This included a reduction in (i) TNF-*α*, IL-1*β*, and iNOS expression, (ii) NF-*κ*B activation, (iii) neutrophil infiltration, (iv) nitrotyrosine and lipid peroxidation, (v) FasL expression, and (vi) TUNEL staining [23]. In accord with these findings, data demonstrate that GW0742 administered 1 hour prior to SCI reduced cell death and inflammation in cultured murine spinal cord slices. The reduction in inflammation included decreased (i) Cox-2 expression and (ii) p38 MAPK, c-Jun N-terminal kinase, and NF-*κ*B activation [88].

It has also been proposed that PPAR*δ* attenuates SCI by promoting oligodendrocyte precursor cell (OPC) proliferation and differentiation following injury. Studies have shown that OPCs accumulate at SCI sites, likely as an attempt to regenerate the damaged axons [89]. Following SCI in rats, PPAR*δ* positive cells increased in the white matter along the lesion border for up to two weeks. Double-labeling experiments indicated there was an increase in PPAR*δ* in NG2 positive cells, potential OPCs, and PPAR*δ* positive CC1 cells, oligodendrocytes [90]. Given the temporal and spatial expression of PPAR*δ* after SCI, it is possible that it can regulate oligodendrogenesis after injury. 

## 10. Summary

Taken together, the findings described above indicate that PPAR*δ* agonists show promise as potential therapeutic agents for a variety of CNS pathologies, including stroke, MS, AD, PD, radiation-induced brain injury, and SCI. The neuroprotective efficacy of PPAR*δ* agonists are, in large part, mediated by modulating oxidative stress and inflammatory processes; the neuroprotective benefits of PPAR*δ* agonists are summarized in Table 1. Future studies using *in vitro* and *in vivo* approaches should further address the mechanisms by which PPAR*δ* exerts its antioxidant and anti-inflammatory effects and help establish the foundations for future clinical applications of PPAR*δ* agonists.

## Figures and Tables

**Table 1:** The neuroprotective benefits of PPAR*δ* agonists.

CNS disorder	PPAR*δ* agonist	Neuroprotective benefits
Stroke	GW501516	Significantly attenuated the cortical infarct area following MCAO-induced ischemic damage in rats [12]
		Reduced OGD-induced MMP-9 enzymatic activity in mice [13]
		Protected against vascular endothelial cell death by inhibiting expression of miR-15a, which increased Bcl-2 expression, reduced golgi fragmentation, and decreased caspase-3 activity [43]
	L-165041	Significantly attenuated the cortical infarct area following MCAO-induced ischemic damage in rats [12]

Multiple sclerosis	GW0742	Reduced emergence of new cortical lesions, increased PLP mRNA, and reduced IL-1*β* expression in mice immunized with MOG [14]
	GW501516	Reduced IL-1, IL-23, IFN*γ*, and IL-17 and increased IL-4 and IL-10 in mice immunized with MOG [50]
		Reduced LPS-induced TNF-*α* and iNOS mRNA in aggregating brain cells [54]
	L-165041	Reduced IL-1, IL-23, IFN*γ*, and IL-17 and increased IL-4 and IL-10 in mice immunized with MOG [50]

Alzheimer's disease	GW0742	Blocked A*β* peptide-induced cell death, as measured by LDH release and Fluorojade B staining, in primary cultures of rat cortical neurons [21]
		Decreased amyloid burden, increased neprilysin expression, and decreased GFAP expression in 5xFAD mice [22]
	L-160,043	Prevented STZ-induced neurodegeneration and cognitive impairment in rats. This was associated with reduced tau phosphorylation, increased choline acetyltransferase, attenuated 8-OHdG and HNE, and reduced GFAP expression [15]

Parkinson's disease	GW501516	Ameliorated MPTP-induced depletion of striatal dopamine and its metabolites in mice [12]
		Reduced Caspase-3 and protected SH-SY5Y cells from MPP+-induced cell death [12]
	L-165041	Ameliorated MPTP-induced depletion of striatal dopamine and its metabolites in mice [12]
		Reduced Caspase-3 and protected SH-SY5Y cells from MPP+-induced cell death [12]

Radiation-induced brain injury	GW0742	Prevented microglial activation (CD68+ cells) in mice irradiated with a single dose of 10 Gy (unpublished)
	L-165041	Prevented intracellular ROS generation, Cox-2, iNOS, and MCP-1 expression, IL-1*β* and TNF-*α* mRNA, and NF-*κ*B, and AP-1 activation in BV-2 cells irradiated with 10 Gy (unpublished)

Spinal cord injury	GW0742	Improved motor function of mice and reduced TNF-*α*, IL-1*β*, and iNOS expression, NF-*κ*B activation, neutrophil infiltration, nitrotyrosine and lipid peroxidation, FasL expression, and TUNEL staining [23]
		Reduced Cox-2, p-p38 MAPK, and p-c-jun expression, and NF-*κ*B activation in murine spinal cord slices [88]
